# Arthroscopic Treatment of 2 Consecutive Cases of Dysplasia Epiphysealis Hemimelica of the Ankle: A 5-Year Follow-Up Report

**DOI:** 10.1155/2017/3175765

**Published:** 2017-05-30

**Authors:** Cosma Calderaro, Carlo Iorio, Francesco Turturro, Federico Morelli, Luca Labianca, Antonello Montanaro, Andrea Ferretti

**Affiliations:** Orthopaedic and Traumatology Department, S. Andrea Hospital, Faculty of Medicine and Psychology, “Sapienza” University of Rome, Rome, Italy

## Abstract

The dysplasia epiphysealis hemimelica (DEH) is a rare disease of unknown etiology consisting in an abnormal osteocartilaginous growth at the epiphysis, usually hemimelic with histological findings similar to benign osteochondroma. In this case series, we described the results of the arthroscopic treatment of 2 consecutive cases of intra-articular ankle localization of DEH in 2 patients aged 9 and 10 years. The good result obtained, persistent at the 5-year follow-up, leads us to consider the arthroscopic approach as a reliable treatment in patient affected by intra-articular ankle DEH.

## 1. Introduction

Dysplasia epiphysealis hemimelica (DEH), firstly reported by Muchet and Berlot in 1926 and called tarsomegaly [1], was better described by Trevor in 1950 [2] (Trevor's disease) and Fairbank in 1956 [3]. It is a rare disorder of unknown etiology with an estimated incidence of 1 : 1,000,000, with a male to female ratio of 3 : 1 [4–6].

Generally diagnosed between 2 and 14 years of age, it consists of an abnormal osteocartilaginous growth localized at the epiphysis, usually hemimelic (affecting the medial side more likely than the lateral) [2], with histological findings that can be similar to benign osteochondroma [3]. The DEH appears to be nonhereditary, and the lesions are not premalignant [7, 8] and frequently localized at lower limbs, especially ankle and knee (79% of cases) [9], even though cases have also been reported in the acetabulum, carpal bones, shoulder, wrist, calcaneus, and patella [10].

Azouz classified the DEH in 3 types: localized, when a single epiphysis (usually in the hind foot or ankle) is involved; classic, when more than one epiphysis of the same limb (2/3 of cases) is involved; generalized, when a whole limb is involved [11].

Histologically, however, the lesions are similar to osteochondroma; DEH lacks the EXT1 and EXT2 gene mutations typical of the osteochondroma [12, 13]. The lesions of the DEH continue to grow until skeletal maturity and an early physeal plate closure can be associated [14]. The intra-articular lesions can be complicated, with recurrence and deformities [6]. Malignant transformations are not described to our best knowledge.

DEH treatment is controversial because the rarity of the disease and absence of high level study. Asymptomatic lesions can need only clinical observation [15], while the surgical removal is very common when symptomatic [16].

## 2. Cases Presentation

### 2.1. Case  1

**Figure 1:**
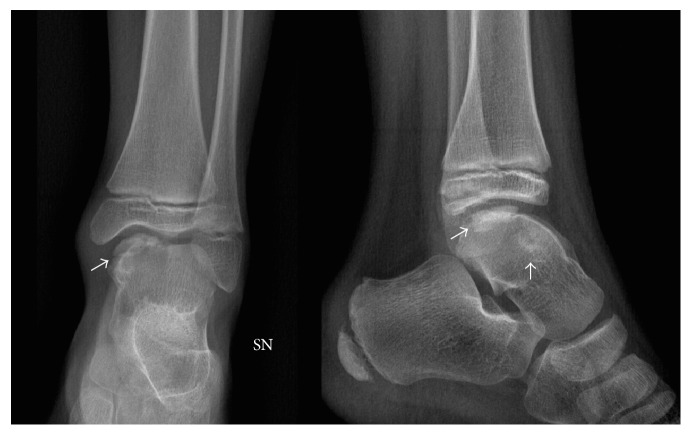
The X-rays show a bony-like protuberance localized at the anteromedial and posteromedial aspect of the talus (arrows).

**Figure 2:**
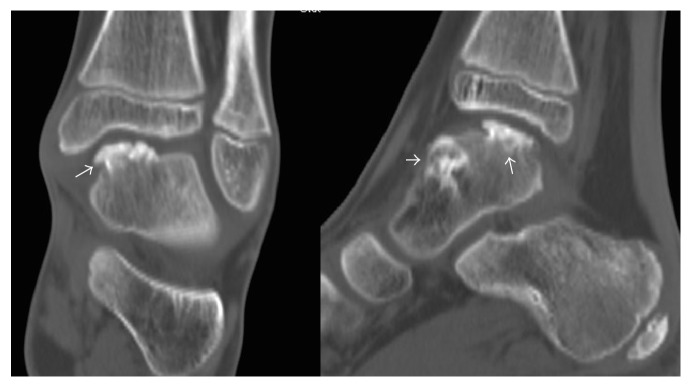
The CT scan shows the bony-like protuberances arising at the anteromedial and posteromedial aspect of the talus (arrows) and the relationships with the other bone structures of the ankle.

**Figure 3:**
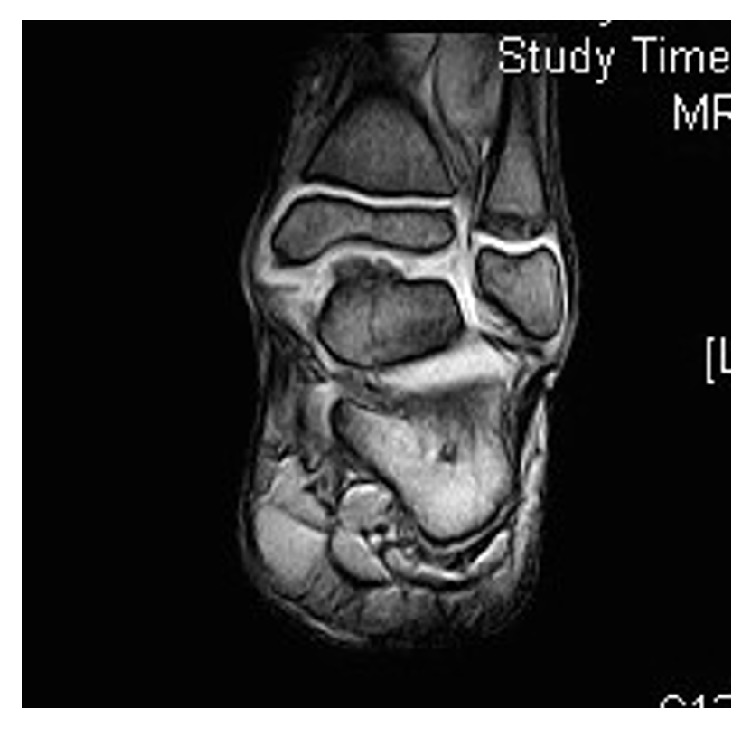
MRI view of the lesion.

**Figure 4:**
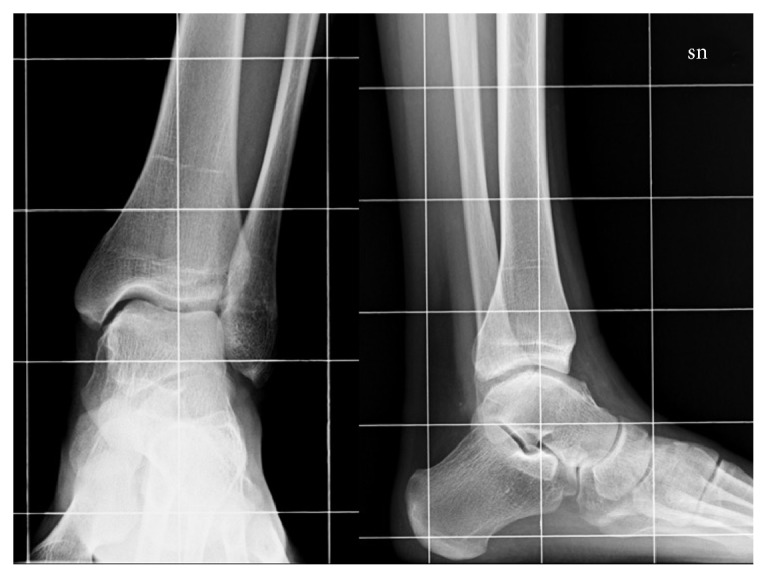
Five-year follow-up X-rays without signs of recurrence or arthritis.

In July 2010, a 9-year-old Caucasian male presented to our attention for persistent left ankle pain that began 40 days before following an ankle sprain playing soccer. The pain was not present during night but occurred with sport activities and walking for long time. The clinical examination showed pain in the anteromedial and posteromedial region of the ankle without remarkable soft tissue swelling and a slight reduction (5°–10°) of the dorsiflexion in comparison to the contralateral ankle; there was no limping and the stability tests of the ankle were negative. Plain radiographs showed bony-like irregular protuberances localized at the anteromedial and posteromedial aspect of the talus (Figure 1). Computed Tomography (CT, Figure 2) and Magnetic Resonance Imaging (MRI, Figure 3) were performed and they were useful to confirm findings consistent with Trevor's disease lesions. Because the young age, the first treatment approach was a rehabilitation program consisting in proprioceptive exercises, strengthening of calf muscle, physical therapy, and Ibuprofen 200 mg when needed. The outpatient examination after 1 month and 6 months did not show any improvement of the symptoms; then, in September 2011, an arthroscopic removal of the posteromedial protuberance was performed by a posterior two-portal approach (Figure 4); the anteromedial exostoses were removed by an open surgical approach during the same session. After surgery the left ankle was protected with an elastic-compressive bandage for 2 weeks and active ROM recovery was encouraged. Progressive weight bearing on the left limb was allowed starting from the third week and the full weight bearing without crutches was achieved in 2 weeks. The outpatient clinic examinations were performed at 2 weeks, 4 weeks, and 3 months after surgery, when the patient was allowed to return to his daily and sport activities. At the 6-month follow-up, there was no recurrence of pain or any other issues. No perioperative and postoperative complications were recorded.

**Figure 5:**
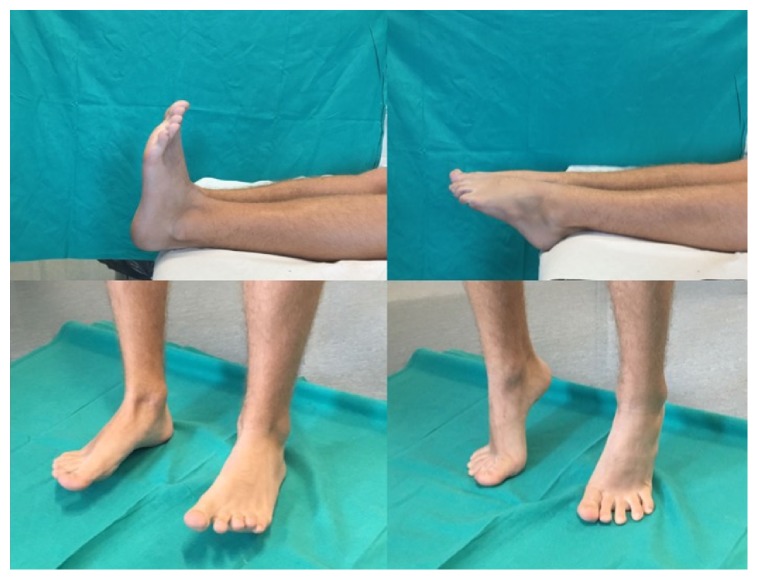
Clinical examination at the 5-year follow-up.

**Figure 6:**
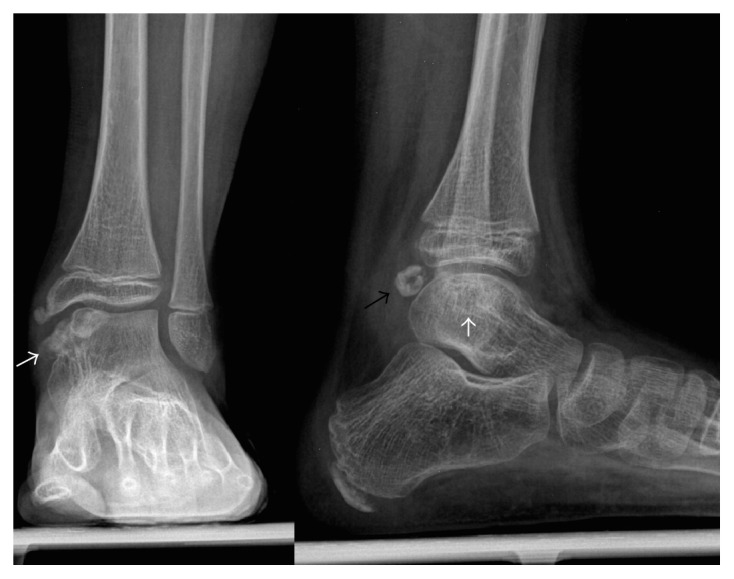
X-rays show medial talus and tibia bony formations (white arrows) and posterior cauliflower fashioned loose body (black arrow).

The patient was recalled in October 2016 (5 years after surgery) for a clinical and radiographic examination. The X-rays showed that the left ankle was free of recurrence and there were not ankle arthritis signs (Figure 5). The clinical examination showed that the left ankle was stable, no limping, ROM similar to the contralateral ankle, absence of pain, and numbness (Figure 6). The patient recovered his full daily and sport activities. He and his parents were satisfied with the result of the procedure.

### 2.2. Case  2

In January 2011, a 10-year-old Caucasian male referred to our hospital for a 3-month ongoing story of pain and swelling of the medial aspect of the left ankle. There was no history of trauma, fever, other joints pain, and abnormalities. He experienced pain walking and playing sports, with limping at the end of the day. Clinically, the ankle joint was painful at the medial edge from the anterior to the posterior aspect. Both dorsiflexion and plantar flexion were reduced of 10°, with local swelling and mild warmth.

**Figure 7:**
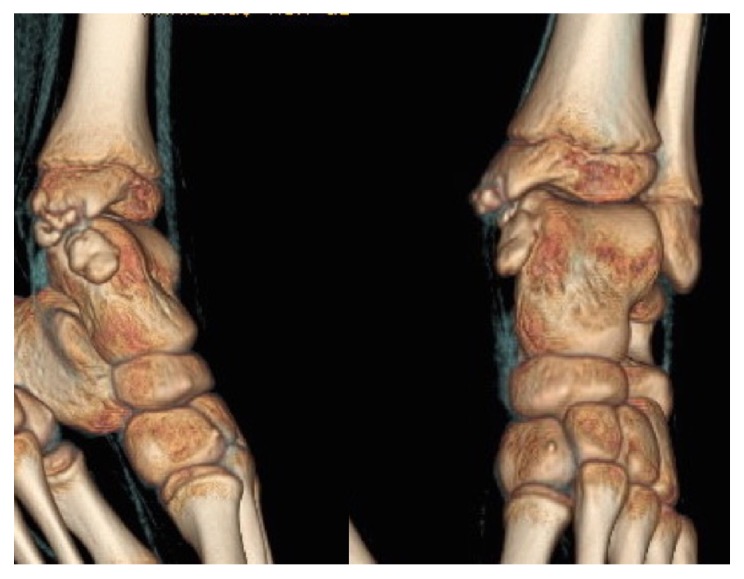
3D reconstruction CT scan showing medial lobulated bony prominences.

**Figure 8:**
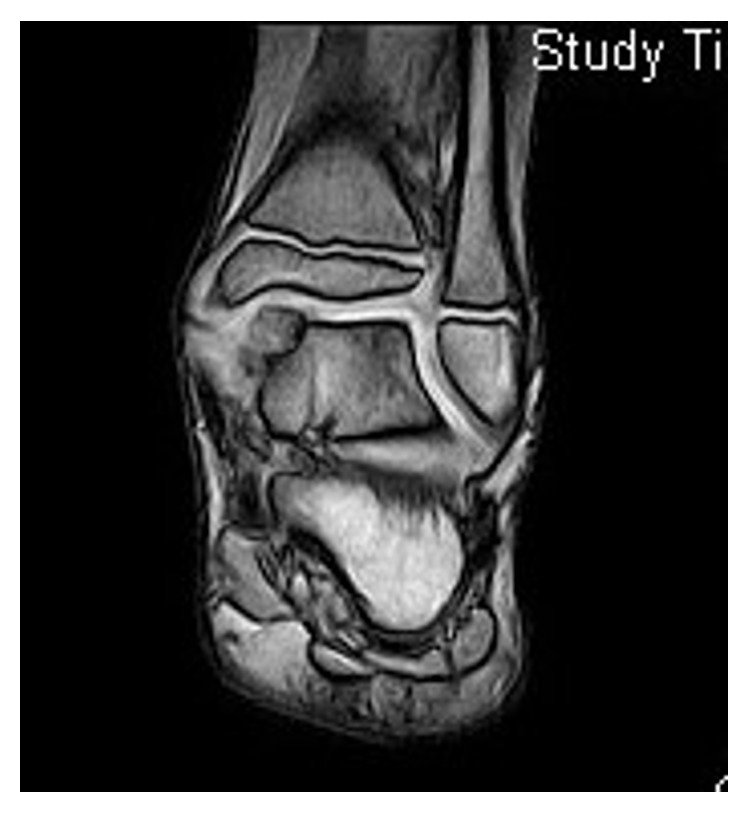
The MRI shows pedicled bone tumor arising from the talus.

X-rays examination showed a protuberance of bony-like nature starting from the medial articular portion of the talus and with lobulated appearance and a large intra-articular cauliflower fashioned loose body (Figure 6). The 3D reconstruction CT scan demonstrated an expansion of the bone arising from the anteromedial aspect of the talus associated with an irregularity of the distal third of the tibia (Figure 7); MRI described a pedicled oval bone tumor arising from the talus (Figure 8).

Because of the symptoms described and the imaging evidences, the first choice of treatment was the surgical arthroscopic removal by a posterior two-portal approach. The arthroscopy showed a flush reactive synovitis that was removed using the motorized tool. The intra-articular loose body was completely removed. The large pedicled prominence was arthroscopically detected but because of the need to perform the histological examination, the removal was performed by an open anteromedial approach. The rehabilitation program was the same as the previous case described. The histological examination showed a cartilaginous and bony-like mass without any evidence of neoplastic cells; then the clinical and pathological diagnosis was DEH of the ankle. The outpatient examinations were performed 2 weeks, 4 weeks, and 3 months after surgery, when the return to his days and sport activity was allowed. At the 6-month follow-up, there was no recurrence of pain and swelling or any other issues and there were no evidences of any perioperative and postoperative complication.

**Figure 9:**
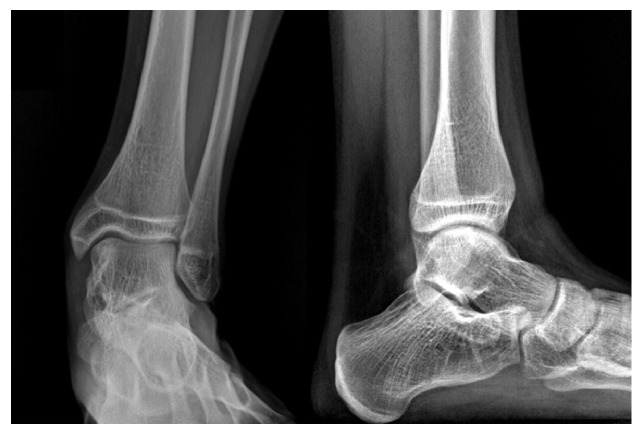
Five-year follow-up X-rays without signs of recurrence or arthritis.

**Figure 10:**
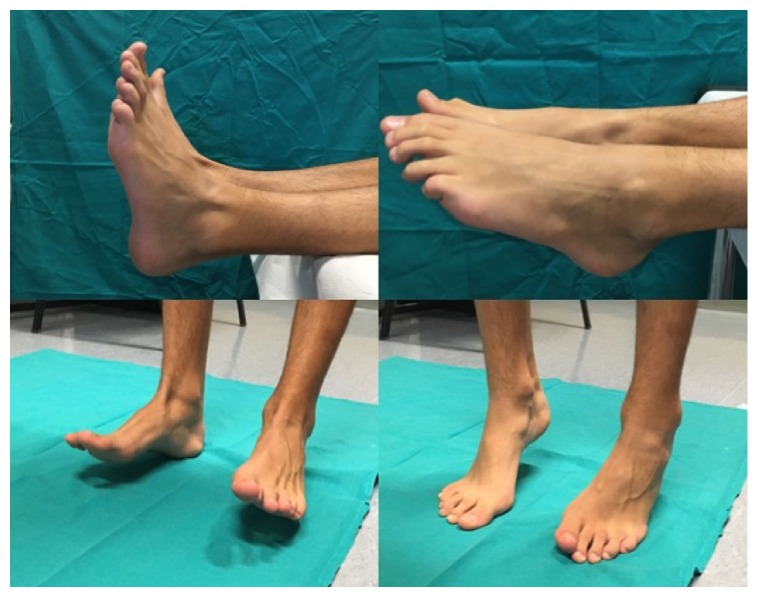
Range of motion and clinical findings at the 5-year follow-up.

The patient was recalled in October 2016 (5 years after surgery) for a clinical and radiological examination. The X-rays (Figure 9) did not show signs of recurrence and there were no signs of ankle arthritis. The clinical examination showed that the ankle was stable; the ROM limitation was less than 5° in comparison with the contralateral without clinical significance; there was absence of pain and swelling (Figure 10). The patient returned to his previous level of sport activity without restrictions. He was very satisfied and there were no other concerns.

## 3. Discussion

The aim of this paper was to describe the good results obtained, in a 5-year follow-up examination, in the treatment of two cases of symptomatic DEH of the ankle by the arthroscopic approach.

The first patient referred to pain after a first-grade ankle sprain with symptoms that were refractive to a prolonged physical therapy and anti-inflammatory drugs. The second case was a patient with an atraumatic ankle pain, with swelling and reactive synovitis.

Despite the different presentation, in both cases the arthroscopic removal of the lesions restored a good function with results that lasted after 5 years.

The arthroscopic approach to these kind of lesions seems to be growing in the literature, although the rarity of the condition does not allow us to draw definitive conclusions. In a recent accurate review of the literature, Gökkuş et al. suggested that carrying out excisions is a better choice because of a large number of good results, although the number of cases in the literature is limited. They highlighted the chance to treat these lesions by an arthroscopic approach [17]. Previously, the same author reported an interesting case report in which a DEH lesion mimed an anterior ankle impingement syndrome. The patient was treated with the arthroscopic excision of the anterior tibial protuberance that appeared like an osteochondromatous lesion, and the nature was confirmed later by the histopathologic examination. The author reported early good result of the procedure with disappearance of the pain and of the limitation of range of motion within the first postoperative week [18].

An important issue that has to be highlighted is the role of the MRI in the diagnosis of this condition. Although the traditional radiological images provide some important information for the diagnosis, the MRI examination is extremely useful for the differential diagnosis, in identifying the extension of the epiphyseal lesion, and in identifying the eventual joint deformity and the soft tissue changes associated with DEH. Also, the role of MRI is important both in the follow-up and in the recurrence [19].

After an accurate review of the literature and after our good experience, because the natural history of the lesion is to grow until skeletal maturity, when localized intra-articular, the removal of the osteochondromatous lesions should be performed arthroscopically, although asymptomatic, to avoid the risk of articular cartilage degeneration and early osteoarthritis [20].

In conclusion, the arthroscopic removal of the ankle lesions can be considered a viable option for the DEH treatment.
